# A promising predictive biomarker combined EBV NDA with PNI for nasopharyngeal carcinoma in nonendemic area of China

**DOI:** 10.1038/s41598-023-38396-z

**Published:** 2023-07-20

**Authors:** Qiao He, Yecai Huang, Linjia Yuan, Zuo Wang, Qiuju Wang, Daduan Liu, Luona Li, Xianbing Li, Zhi Cao, Dongsheng Wang, Mu Yang

**Affiliations:** 1 School of Medicine, University of Electronic Science and Technology of China, Chengdu, China; 2 Department of Clinical Laboratory, Sichuan Clinical Research Center for Cancer, Sichuan Cancer Hospital & Institute, Sichuan Cancer Center, Affiliated Cancer Hospital of University of Electronic Science and Technology of China, No. 55, South Renmin Road, Chengdu, 610041 China; 3 Department of Radiation Oncology, Radiation Oncology Key Laboratory of Sichuan Province, Sichuan Clinical Research Center for Cancer, Sichuan Cancer Hospital & Institute, Sichuan Cancer Center, Affiliated Cancer Hospital of University of Electronic Science and Technology of China, No. 55, South Renmin Road, Chengdu, 610041 China; 4 Department of Radiation Oncology, Jinjiang Da Guan Hospital of Chengdu, Chengdu, China; 5 Centre for Translational Research, Sichuan Clinical Research Center for Cancer, Sichuan Cancer Hospital & Institute, Sichuan Cancer Center, Affiliated Cancer Hospital of University of Electronic Science and Technology of China, Chengdu, China

**Keywords:** Head and neck cancer, Microbiology, Molecular biology, Biomarkers

## Abstract

In endemic areas, EBV DNA is used to guide diagnosis, detect recurrence and distant metastasis of NPC. Until now, the importance of EBV DNA in the prediction of NPC has received little attention in non-endemic regions. To explore the prognostic value of EBV DNA alone or in combination with PNI in NPC patients from a non-endemic area of China. In this retrospective study, 493 NPC patients were enrolled. Clinical pathologic data, pre-treatment plasma EBV DNA, and laboratory tests were all performed. A standard anticancer treatment was prescribed, and follow up data were collected. EBV DNA was found to be positively related to clinical stage (r = 0.357, *P* < 0.001), T stage (r = 0.193, *P* < 0.001), N stage (r = 0.281, *P* < 0.001), and M stage (r = 0.215, *P* < 0.001). The difference in EBV DNA loads between clinical stage, T, N and M stage was statistically significant (*P* < 0.001). In this study, the best cutoff value for EBV-DNA to distinguish the prognosis of NPC was 262.7 copies/ml. The 5-year OS of patients in the EBV-DNA ≤ 262.7 copies/ml group and EBV-DNA > 262.7 copies/ml group was 88% and 65.3%, respectively (*P* < 0.001). EBV-DNA and PNI were found to be independent prognostic factors for OS in multivariate analysis (*P* < 0.05). EBV-DNA was independent prognostic factors for PFS. In predicting NPC patients OS, the novel combination marker of EBV DNA and PNI outperformed TNM staging (AUC: 0.709 vs. 0.675). In addition, the difference between EBV + PNI and EBV + TNM was not statistically significant for OS or PFS (*P* > 0.05). This novel combination biomarker was a promising biomarker for predicting NPC survival and may one day guide treatment option.

## Introduction

Nasopharyngeal carcinoma (NPC) is frequent in east and southeast Asia worldwide^1^.People with high prevalence of NPC in the Chinese population are mostly concentrated in the country’s south^2^. Guangxi, Guangdong and Hunan were the top three most prevalent provinces, with incidence more than three times higher than that in nonendemic areas^2^. NPC is also an Epstein–Barr virus (EBV) related malignant tumor, people living in endemic areas are more likely to develop NPC due to infection with non-synonymous EBV variants within BALF2^3^. A large sample meta-analysis revealed that circulating EBV DNA predicted NPC survival in southeast Asia’s endemic area. Patients with NPC who had higher EBV DNA levels had a lower chance of survival^4^. EBV DNA was also used to guide diagnosis, detect recurrence and distant metastasis of NPC in the endemic area^5,6^. Until now, the value of EBV DNA in the diagnosis and prediction of NPC in patients living in non-endemic regions received little attention.

We have explored the diagnostic value of EBV DNA for NPC in three non-endemic hospitals, which showed that NPC patients had higher pre-treatment EBV DNA load, the diagnostic sensitivity could reach 80.5%, and the area under curve (AUC) is 0.901 if EBV DNA was used as a biomarker to detect NPC (baseline = 0 copies/ml)^7^. This finding was consistent with the data from other countries’ endemic and non-endemic regions^8,9^. However, due to differences in sample type, DNA isolation protocol, PCR assay of different institutions and the absence of international practice guidelines to detect EBV DNA, the prognostic value of EBV DNA in China’ non-endemic region have not been reported.

In addition to EBV DNA, the prognostic nutritional index (PNI) is widely accepted biomarker for predicting NPC survival^10–12^. In 2019, we reported that a novel prognosis index of PNI combined with age could predict survival of locally advanced NPC who received neoadjuvant chemotherapy plus concurrent chemoradiotherapy^10^. PNI can reflect treatment tolerance to anticancer treatment, predict overall survival (OS) and progression free survival (PFS). Recently, Rong Zhao et.al^11^ also found that the integrating inflammatory biomarkers and nutritional indicators could predict OS of locally advanced NPC patients. However, no one has investigated the prognostic value of the combination biomarker of EBV DNA and PNI in endemic region or non-endemic region.

In this study, we first explored the prognostic value of EBV DNA for NPC in non-endemic area. Following that, we constructed the novel combination biomarker with EBV DNA and PNI in NPC patients.

## Results

### Clinical pathology characteristics of patients

A total of 493 NPC patients were enrolled in this study. The median age of the patients was 50 years (range, 43–60 years). 346 (70.2%) of the 493 patients were male, while 147 (29.8%) were female. Table 1 summarized the clinical stage (restaged according to AJCC/UICC 8th), pathology type, history of alcoholism, smoking history, family history of cancer or NPC, PNI and EBV DNA. The expression of Epstein-Barr virus-encoded small RNA (EBER) on tumor tissue revealed the virological assessment of EBV. EBER expression was found in 256 patients, with 243 (243/256, 94.9%) expressing it positively.

**Table 1:** Basic characteristics of patients enrolled in this study.

Characteristics	N(%)	Characteristics	N(%)
Age(y)	50(43,60)	Pathology type	
Gender		Non-keratinizing carcinoma	478(96.96)
Male	346(70.2)	Keratinizing carcinoma	15(3.04)
Female	147(29.8)	Alcohol	
T stage*		Yes	150(30.43)
T1	29(5.88)	No	343(69.57)
T2	128(25.96)	Smoking	
T3	166(33.67)	Yes	210(42.60)
T4	170(34.48)	No	283(57.40)
N stage*		Family history of cancer	
N0	12(2.44)	Yes	55(11.16)
N1	74(15.01)	No	438(88.84)
N2	291(59.02)	Family history of NPC	
N3	116(23.53)	Yes	17(3.45)
M stage*		No	476(96.55)
M0	456(92.49)	PNI	
M1	37(7.51)	≤ 52.6	304(61.70)
Clinical stage*		> 52.6	189(38.30)
II	29(5.88)	EBV DNA(Copies/ml)	
III	200(40.57)	≤ 262.7	317(64.30)
IVa	227(46.04)	> 262.7	176(35.70)
IVb	37(7.51)		

### Correlation between plasma EBV DNA and various stages and pathology types

Although EBV DNA was positively correlated with clinical stage (r = 0.357, *P* < 0.001), T stage (r = 0.193, *P* < 0.001), N stage (r = 0.281, *P* < 0.001), and M stage (r = 0.215, *P* < 0.001) of NPC patients, there was no significant correlation between EBV DNA and pathology type, history of alcoholism, smoking, family history of cancer and NPC (*P* > 0.05).

The difference in EBV DNA loads between clinical stage, T stages, N stages and M stages was statistically significant (*P* < 0.001, Table 2). No significant difference was observed in pathology type subgroups (*P* = 0.090, Table 2).

**Table 2:** Difference of EBV DNA loads between each subgroup.

	EBV-DNA(N)	K	*P*
Clinical stage		63.68	< 0.001
II	0(0,121.13)		
III	41.04(0,255.82)		
IVa	167.57(41.7,1096.00)		
IVB	1380.00(111.90,4220.00)		
T stage		18.92	< 0.001
T1	40.9(0,167.35)		
T2	53.7(0,523.00)		
T3	77.42(14.98,658.75)		
T4	173.56(43.72,1095.00)		
N stage		43.14	< 0.001
N0	0(0,68.59)		
N1	45(0,275.5)		
N2	95.6(16.44,433.58)		
N3	530(56.18,2707.5)		
M stage		22.65	< 0.001
M0	95.03(14.41,541.5)		
M1	1280(152.64,4270.00))		
Pathology type		2.878	0.090
Keratinizing carcinoma	107.80(16.86,676.25)		
Non-keratinizing carcinoma	30.54(0,315.54)		

### Relationship between EBV DNA and prognosis of NPC in nonendemic area

Prior to treatment, 103 out of 493 patients (20.89%) in this study had undetectable plasma EBV DNA . The median EBV DNA value was 101.85copies/ml (ranged, 0–1.04*10^6^copies/ml), the first quartile was 16.35 copies/ml, and the third quartile was 658.75 copies/ml. All patients were divided into four groups: 0–25% was P1, 25–50% was P2, 50–75% was P3, and 75–100% was P4. EBV DNA levels in the P1, P2, P3, P4 groups were 0–16.35 copies/ml (N = 123), 16.35–101.85 copies/ml (N = 124), 101.85–658.75 (N = 123), greater than 758.75 copies/ml (N = 123), respectively.

**Figure 1:**
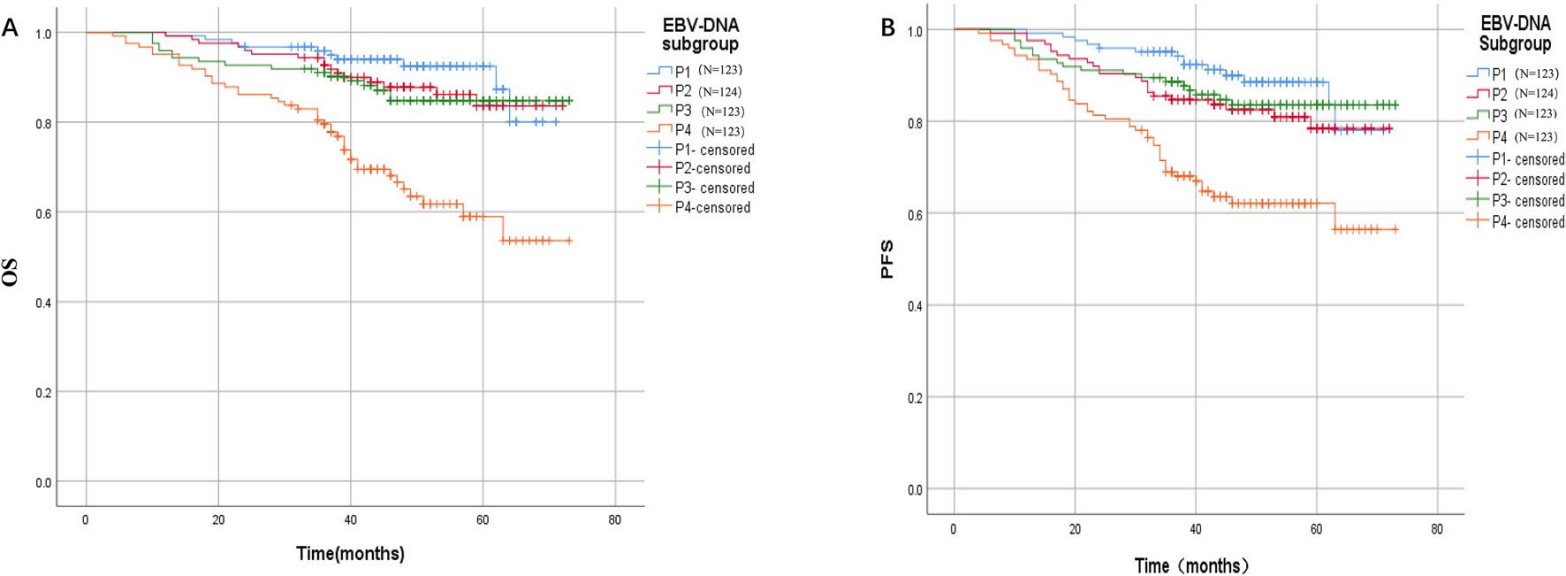
Survival curve of patients with different EBV DNA loads. (**A**) Overall survival of patients with different plasma EBV DNA loads. (**B**) Progression free survival of patients with different plasma EBV DNA loads. P1 EBV DNA 0–16.35 copies/ml (N = 123), P2, EBV DNA 16.35–101.85 copies/ml (N = 124), P3 EBV DNA 101.85–658.75 Copies/ml (N = 123), P4 EBV DNA > 658.75 copies/ml.

The median follow-up of this study was 47 months (4–73 months). Survival analysis and comparison of survival rates between groups were depicted in Fig. 1. The 5-year 0S of P1, P2, P3, and P4 groups were 92.4%, 83.6%, 84.8%, and 58.9%, respectively. The survival difference between P4 and P1, P2, and P3 groups were statistically significant (*P* < 0.001). There was no significant difference in OS among the remaining subgroup groups (*P* > 0.05). The P1, P2, P3, and P4 groups had 5-year PFS of 88.5%, 78.4%, 83.5%, and 62.0%, respectively. The difference in survival between P4 and P1, P2, and P3 group were statistically significant (*P* < 0.001, 0.001 and < 0.001). There was no significant difference in PFS among the remaining subgroup groups (*P* > 0.05).

### Exploration of the best EBV DNA cutoff value to differentiate the prognosis of NPC in nonendemic area

**Figure 2:**
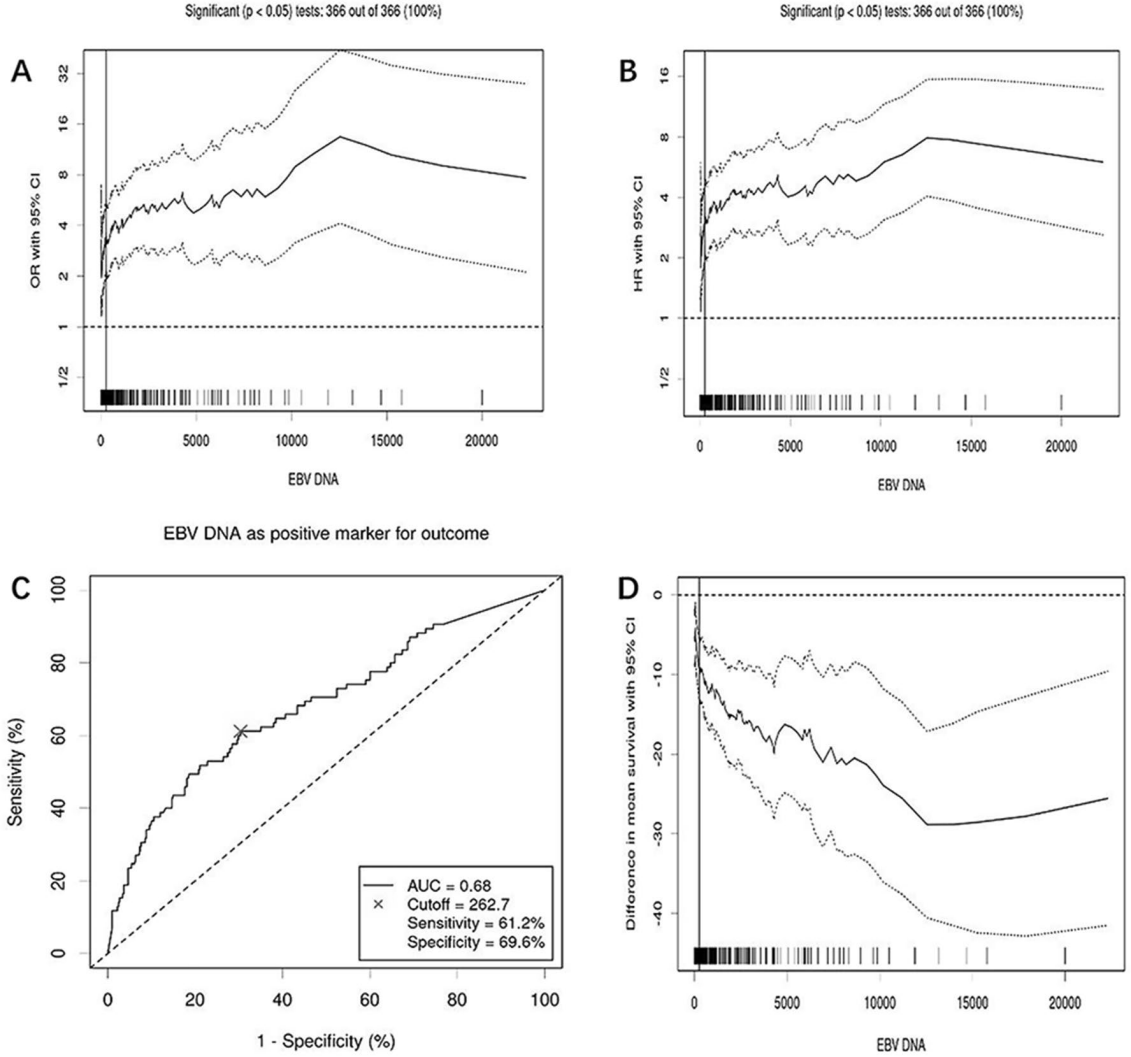
Cutoff value of EBV-DNA for OS. (**A**) OR with 95% CI of EBV DNA as a biomarker for OS. (**B**) HR with 95% CI of EBV DNA as a biomarker for OS. (**C**) ROC curve of EBV DNA as a biomarker for OS. D. Difference in mean survival with 95% CI among NPC with different plasma EBV DNA loads.

**Figure 3:**
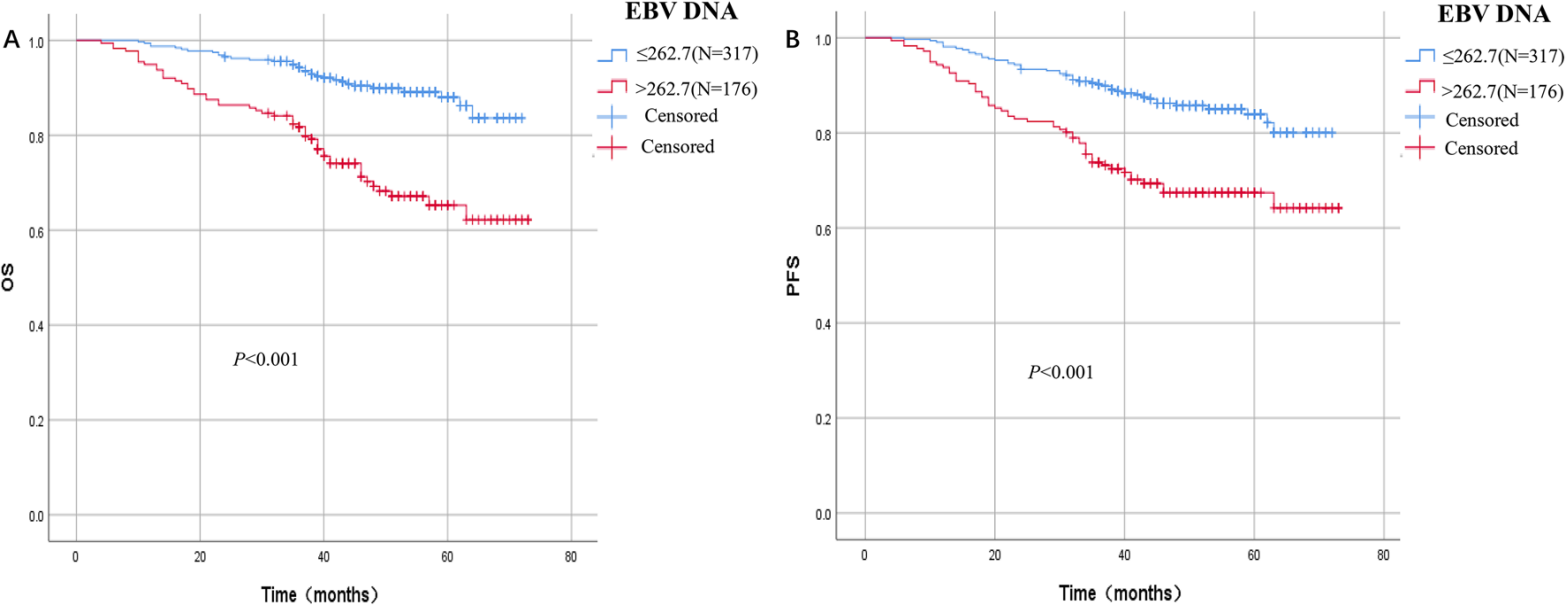
Survival curve of patients with different EBV DNA loads. (**A**) Overall survival of patients with different plasma EBV DNA loads. (**B**) Progression free survival of patients with different plasma EBV DNA loads.

The EBV DNA and OS of NPC patients were uploaded to the cutoff finder (https://molpathoheidelberg.shinyapps.io/CutoffFinder_v1/), and the ROC curve (Euclidean distance) method was used to differentiate the best cutoff value of EBV-DNA for prognosis. The cutoff value for OS was 262.7 copies/ml, and the HR was 3.3 (2.13–5.1). The ROC curve’s AUC was 0.68, its sensitivity was 61.2%, and its specificity was 69.6%. The 5-year OS in the EBV-DNA ≤ 262.7copies/ml (N = 317) and > 262.7 copies/ml (N = 176) group were 88% and 65.3%, respectively (*P* < 0.001, chi-square 32.12, Fig. 2). The 5-year PFS of EBV-DNA ≤ 262.7 copies/ml group (N = 317) and EBV-DNA > 262.7 copies/ml (N = 176) were 83.9% and 67.4%, respectively, and the difference was statistically significant (*P* < 0.001, chi-square value 22.07) (Fig. 3). In addition, subgroup analysis was performed to compare the prognosis of EBV-DNA ≤ 262.7copies/ml group and EBV-DNA > 262.7 copies/ml group in locally advanced stage (LA, N = 428), M0 (N = 456), and M1 (N = 37) patients, respectively. In the LA, M0, and M1 stage, the 5-year OS of patients with EBV-DNA ≤ 262.7 copies/ml was 88.0%, 88.9%, and 82.9%, respectively, while the EBV-DNA > 262.7 copies/ml group was 69.5%, 70.2%, and 56.8% (Figure S1A,C,E, all *P* < 0.001). Patients in the EBV-DNA ≤ 262.7 copies/ml group had 83.3%, 84.6%, and 82.9% of 5-year PFS, while those in the EBV-DNA > 262.7 copies/ml group had 72.6%, 73.2%, and 54.6% (Figure S1B, D, F, all *P* ≤ 0.003).

### Univariate and multivariate analysis for OS and PFS

Univariate analysis showed that differences in OS for Age, T stage, N stage, M stage, Alcohol, Smoking, EBV-DNA, PNI, NLR, HGB (g/L)  were statistically significant (*P* < 0.05). The above- mentioned variables were entered into the Cox proportional hazards regression model of survival analysis. According to the multivariate analysis, Age, T stage, and M stage were independent prognostic factors for OS of the patients enrolled in this study (*P* < 0.05). Among the hematological factors, EBV-DNA and PNI were independent prognostic factors for OS (*P* < 0.05). EBV-DNA > 262.7 was a damaging factor for OS (HR = 2.584 (95% CI 1.645–4.049, *P* < 0.001), PNI > 52.62 is a protective factor for OS (HR = 0.480 (95% CI 0.265–0.867, *P* = 0.015) (Table 3).

**Table 3:** Univariate and multivariate analysis for overall survival and PFS.

		OS				PFS			
		Univariate		Multivariate		Univariate		Multivariate	
Variables	N (%)	X^2^	*P*	HR (95% CI)	*P*	X^2^	*P*	HR (95% CI)	*P*
Age		24.67	< 0.001	2.427 (1.428–4.115)	0.001	16.113	0.006	1.873(1.195,2.933)	0.001
≤ 50.5y	236 (47.8)								
> 50.5y	257 (52.2)								
Gender		0.722	0.396			2.752	0.97		
Female	147 (29.8)								
Male	346 (70.2)								
T stage		10.52	0.001	2.298 (1.290, 4.098)	0.005	7.741	0.005	1.815(1.105,2.985)	0.019
T1–2	155(31.44)								
T3–4	338(68.56)								
N stage				1.197 (0.740–1.934)	0.463				
N0-2	377(76.47)	9.661	0.002			8.989	0.003		
N3	116(23.53)								
M stage		51.0	< 0.001	1.964 (1.267, 3.040)	< 0.001	50.297	< 0.001	1.964 (1.267, 3.040)	< 0.001
M0	456(92.49)								
M1	37(7.51)								
Pathology type		0.165	0.684			0.003	0.955		
Keratinizing	15(3.04)								
Non-keratinizing	478(96.96)								
Alcohol		9.277	0.002	1.616 (0.918, 2.841)	0.960	5.808	0.016	1.333(0.802,2.217)	0.268
Yes	151()								
No	342()								
Smoking		8.286	0.004	1.383 (0.791, 2.415)	0.255	6.235	0.013	1.395(0.854,2.294)	0.189
Yes	282()								
No	211()								
EBV-DNA		32.12	< 0.001	2.584 (1.645, 4.049)	< 0.001	22.074	< 0.001	1.97(1.302,2.976)	0.001
≤ 262.7	317(64.3)								
> 262.7	176(35.7)								
PNI		19.04	< 0.001	2.083(1.153,3.773)	0.015	11.02	0.001	1.284 (0.766, 2.155)	0.343
> 52.62	189(38.3)								
≤ 52.62	304(61.7)								
NLR		8.13	0.004	1.408 (0.887,2.237)	0.147	5.120	0.024	1.30 (0.854, 1.981)	0.221
≤ 2.61	253(51.3%)								
> 2.61	240(48.7%)								
=HGB(g/L)		7.768	0.005	0.706(0.445,1.200)	0.139	3.877	0.049	1.255 (0.821, 1.917)	0.294
> 138.5	216(43.8%)								
≤ 138.5	277(56.2%)								
WBC		0.605	0.437			0.121	0.728		
≤ 6.05	237(48.1%)								
> 6.05	256(51.9%)								

Furthermore, the multivariate analysis showed that age, T stage, and M stage were independent prognostic factors for PFS of the patients enrolled in this study (*P* < 0.05). Only EBV DNA was independent prognostic factors for PFS among the hematological factors studied. EBV DNA > 262.7 was a damaging factor for PFS (HR = 1.97(95% CI 1.302–2.976, *P* = 0.001) (Table 3).

### Construction of PNI–EBV DNA integration marker to differentiate the risk of NPC

EBV DNA and PNI were two independent hematological factors associated with OS of NPC. The HR for EBV DNA (EBV DNA > 262.7 copies/ml) was 2.584 (95% CI 1.645–4.049), whereas the HR for PNI (PNI ≤ 52.62) was 2.083 (1.153–3.773), which was similar to the HR for EBV DNA. As a result, we combined EBV DNA and PNI to develop a simple and practical predicting model. Patients with EBV DNA > 262.7 copies/ml or PNI ≤ 52.62 were scored 1, and patients with EBV ≤ 262.7 or PNI > 52.62 were scored 0. The total risk score was calculated by combining the EBV DNA score and PNI score. Patients with two risk factors (total score = 2) were classified as high risk, patients with one risk factors (total score = 1) were classified as intermedial risk, and patients with neither one risk factor were classified as low risk.

**Figure 4:**
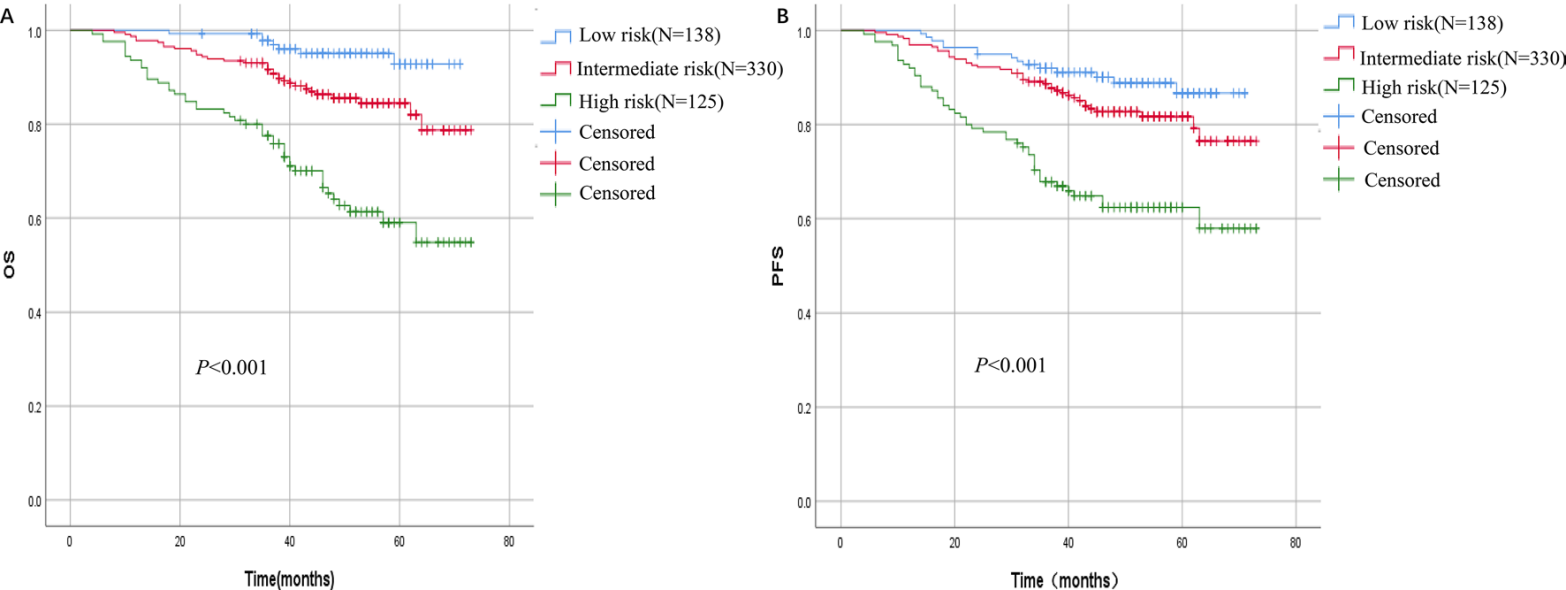
Survival curve of patients with different risk score. (**A**) Overall survival of patients with different risk score. (**B**) Progression free survival of patients with different risk score.

**Figure 5:**
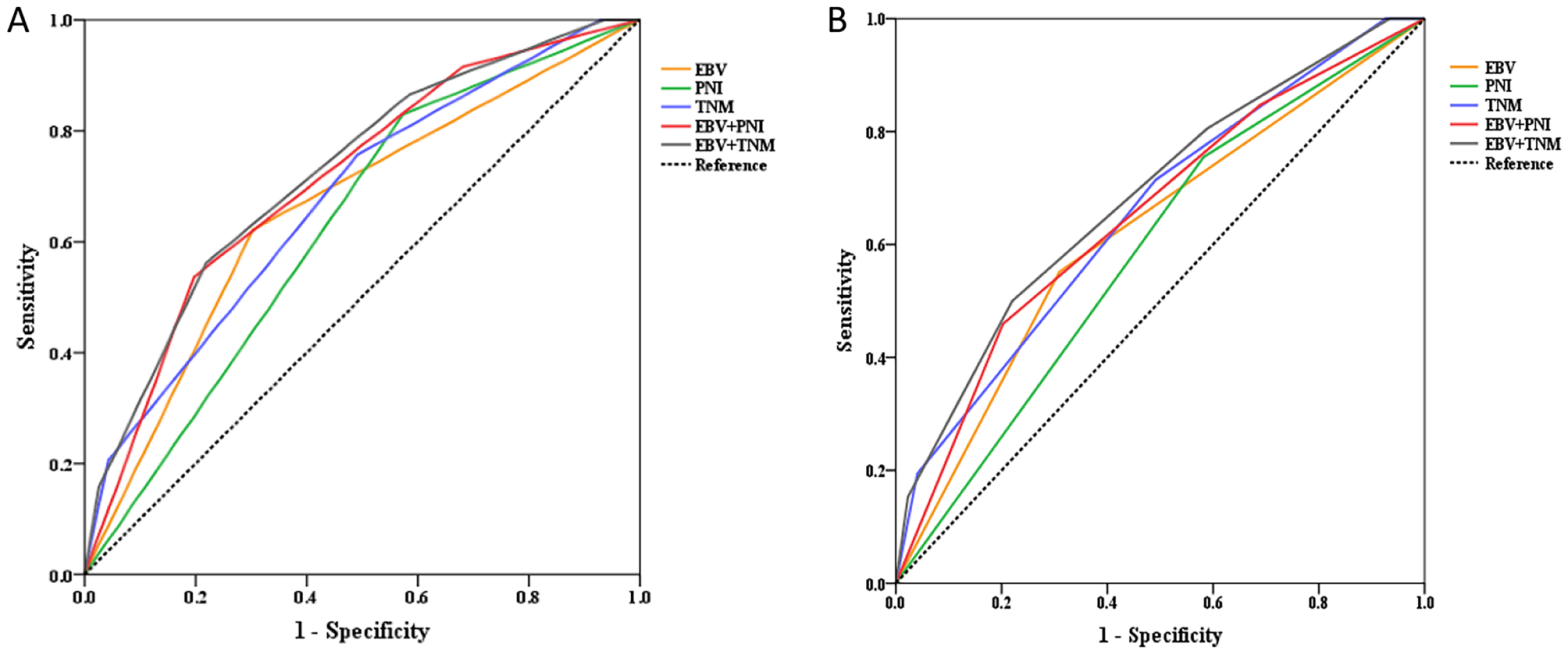
ROC curve of combination model, TNM stage, EBV DNA and PNI for OS and PFS. (**A**) ROC curve of combination model (EBV + PNI/EBV + TNM), TNM stage, EBV DNA and PNI for OS. (**B**) ROC curve of combination model (EBV + PNI and EBV + TNM), TNM stage, EBV DNA and PNI for PFS.

The 5-year OS for low, intermedial and high-risk group was 92.8%, 84.5% and 59% respectively. The difference in survival rate between subgroups was statistically significant (chi square 49.148, *P* < 0.001) The 5-year PFS for low, intermedial and high risk group was 86.7%, 81.75% and 65.9% respectively. The difference in survival rates between subgroups was statistically significant (chi square 33.446, *P* < 0.001) (Fig. 4). We compared the predictive value in different models, including PNI, TNM, EBV, PNI + TNM, EBV + PNI and EBV + TNM (Table S1). For EBV + PNI and EBV + TNM models, the AUC for the OS and PFS were more than 0.7 and 0.6, respectively. The ROC prediction curves for OS and PFS for variables with AUC greater than 0.6 were shown in Fig. 5. The various AUC areas were also statistically analyzed (Table S2), and it was discovered that either EBV + PNI or EBV + TNM performed better than EBV alone (*P* < 0.05), but the difference between EBV + PNI and EBV + TNM was not statistically significant for OS or PFS (*P* > 0.05).

## Discussion

EBV, also known as human herpesvirus 4 (HHV4), is the first human oncogenic DNA virus^13^, infecting over 90% of the world’s population^14^. Nearly 85 genes in the EBV DNA are involved in infection, replication and metabolic reprogramming^15^. Long-term EBV infection has been identified as the primary cause of NPC, EBV-associated gastric carcinoma, EBV-associated B-cell lymphomas, natural killer/T-cell lymphoma, and multiple sclerosis^16–20^.According to the World Health Organization 5th pathology classification, non-keratinizing squamous cell carcinoma (type II) accounted for the greatest proportion of three types of NPC, and this type of NPC is most closely related to EBV infection^16,21^. In this study, 380/478 (79.50%) of patients with WHO type II showed detectable EBV DNA, no significant difference in EBV DNA was observed between type I and type II NPC. Nonetheless, EBV infection is thought to be the leading cause of NPC in both endemic and nonendemic area of China^22^. NPC patients in endemic regions are mainly caused by BALF2, the lytic gene of EBV DNA that is closely associated with viral reactivation and BALF2 gene variation^23^. Plasma EBV DNA, also known as circulating EBV DNA, is made up of naked small viral DNA fragments of 82 base pairs (bp)^24^, which would be detected in 70.3–82.8% of NPC patients in endemic areas^25,26^.

Satoru Kondo found that NPC in non-endemic regions was associated with type 2 EBV or a strain of the BALF2 H-H-L^23^that differed from the endemic strain (the BALF2 H-H-H). In this study, 79.11% patients had detectable plasma EBV DNA, which was consistent with data from the endemic region. However, the cutoff value of EBV DNA for risk segregation differed significantly from the suggested value from the endemic region. Lin et al. suggested a cutoff of 1500 copies/ml as the best predictor of long-term survival^27^. Leung et al. showed that patients with EBV DNA > 4000 copies/ml had significant poorer OS^28^. In a nonendemic region of India, Raja Pramanik et.al proposed a cutoff of 1500 copies/ml to classify patients as having a high or low risk of disease progression or relapse^9^. Our findings showed that the median absolute value of plasma EBV DNA was only 101.85copies/ml, while the suggested cutoff value for survival was 262.7 copies/ml (Fig. 2). Patients with EBV DNA > 262.7 copies/ml had lower survival than those with EBV DNA ≤ 262.7 copies/ml (5-year OS 65.3% vs. 88%, *P* < 0.001, chi-square 32.12, 5-year PFS 67.4% vs. 83.9%, *P* < 0.001, chi-square value 22.07). AS EBV DNA copy number below the detection threshold in some assays was reported as 0 copy/ml or undetectable. Lower detection limit of EBV DNA assays are urgently needed to improve the clinical application value of EBV DNA.

Circulating EBV DNA may provide additional information such as tumor burden, invasive properties and risk of distant metastasis or local recurrence. Adding EBV DNA to the ongoing staging system may improve prognostic performance of the TNM staging, allowing for better treatment options^29–33^. Li WZ et.al showed that the new survival model integrating EBV DNA status and TNM stage outperformed the current TNM stage system^34^. Chen FP et.al built a risk classification system that integrating EBV DNA with T- and N- stage, demonstrating that the novel system outperformed the TNM stage for risk stratification of OS^32^. Sik-Kwan Chan et.al from Hong Kong studied M1 NPC survival by pre-treatment plasma EBV DNA, finding that patients with EBV DNA ≤ 2500 copies/ml reached better OS than those with EBV DNA > 2500 copies/ml (median OS 44.2 vs 19.7 months)^33^.

In this study, plasma EBV DNA was found to be positively correlated with T, N, M and clinical stage of NPC. The difference of EBV DNA in clinical stage, T, N and M categories was statistically significant (Table 2). We then explored the differences in survival between populations based on EBV DNA distribution. P4 patients (EBV DNA > 758.75 copies/ml (N = 123) showed statistically significant worse survival than other groups (Fig. 1). P2 and P3 patients showed similar survival outcomes, and their survival curves followed the same path with some overlap. The heterogeneity of survival curve in P1 group might be explained by the undetectable EBV DNA of 103/123 patients in the P1 group. Some of the patients with undetectable EBV DNA were non-EBV-related NPC or the limitation of detection technology. Further research into the value of including EBV DNA in the staging system in non-endemic regions could improve the application of EBV DNA in NPC clinical practice.

PNI is determined by two value, serum albumin and total lymphocyte count, and was first used to evaluate nutrition and surgical risk in 1980^35^. Until now, three meta-analyses had agreed on the value of PNI to predict NPC patient survival ^12,36,37^, with lower PNI being significantly associated with poor prognosis in NPC. The cutoff value for NPC prognosis ranged from 45.45 to 55^12^. The cutoff value in this study was 52.62, which was in line with our previous study and data reported in other centers ^10,12,36,37^. PNI was widely recognized as a simple tool for assessing cancer patients’ nutrition and immune status. Lower PNI is associated with hypoalbuminemia and/or lymphocytopenia, with hypoalbuminemia usually induced by nutritional issues and lymphocytopenia caused by a weakened immune system. We know that nutrition and immunity can have an impact on each other^38^. Lymphocytopenia is typically associated with immunodeficiency or decreased immunity, which reduces antitumor immunity and the ability to recover from treatment toxicity^39^.

Patients with lower malnutrition had positive relationship with malnutrition, and the malnutrition could lower quality of life and tolerance to anticancer treatment, ultimately increasing chemo-radiotherapy complication and decrease survival^40,41^. Given that 30–50% patients with head and neck cancer were malnourished^42^, nutritional assessment and intervention or treatment have emerged as important aspect of anticancer treatment^43,44^. More attention should be paid to nutritional status in NPC patients during radiotherapy alone or chemoradiotherapy, which could cause malnutrition or worsen pre-existing malnutrition.

Based on the preliminary study on value of EBV DNA and PNI for NPC patients in a nonendemic region of China, we proposed a novel biomarker, EBV DNA-PNI, which was easy to obtain and widely available in cancer centers. Figure 4 showed that the marker performed well in distinguishing the survival risk of NPC patients. On the ROC curve, the AUC of EBV DNA + PNI was greater than OS of TNM staging. In the combination prediction model, either EBV + PNI or EBV + TNM outperformed EBV alone (*P* < 0.05), but the difference for OS or PFS was not statistically significant (*P* > 0.05). These findings suggest that EBV + PNI is a viable predictor of NPC prognosis and has a predictive value for OS that is comparable to the known role of EBV + TNM. As far as we know, this is the first attempt to investigate the combination marker with EBV DNA and PNI to predict NPC survival. Chi Leung Chiang et.al concluded that EBV-DNA level, primary gross tumor volume (GTV), nodal GTV, neutrophil–lymphocyte ratio, C-reactive protein/albumin ratio, platelet count, anemia, lactate dehydrogenase, SUV_max_ of the primary tumor and total lesion glycolysis were the most common non-TNM factors effecting prognosis of NPC in a systematic review^48^. EBV DNA is the most important non-anatomical prognostic factor that could be used to refine the upcoming TNM 9th Edition^45^. However, the suggestion cutoff value for pretreatment EBV-DNA level ranged 1500–25,000 copies/ml, which was much higher than the suggested cutoff value in this study. The disparity could be attributed to a lack of data on non-endemic areas, and the lack of international practice guidelines for EBV DNA detection.

The novel EBV -PNI combination marker included not only EBV DNA, which reflects tumor burden and risk of invasion and metastasis, but also PNI, which reflects patients’ treatment tolerance and immune status. The novel marker outperformed TNM staging and sole EBV-DNA in discriminating OS suggesting that it could be a promising circulation biomarker that could contribute to more precise NPC treatment and staging in the future. This is the first time to systematically report the prognostic value of EBV-DNA in a non-endemic area of China, as well as the combined marker of EBV DNA and PNI to predict NPC prognosis. The following shortcomings could not be avoided. To begin, there are some uncontrollable factors such as EBV DNA detection time point, detection reagent method, and so on that may affect the EBV DNA result. Second, potential selection biases were not avoided because this was a retrospective study. Third, the inconsistent treatment modalities used by the included patients may have an impact on prognosis. Additionally, EBV DNA was quantified with DNA extracted by precipitation concentration method, which may explain the result study’s low cutoff value. Finally, the data in this study are only from a single center, multi-center prospective clinical trials are needed to confirm the prognostic value of EBV DNA in non-endemic areas.

## Conclusion

In non-endemic area, plasma EBV DNA was a prognostic biomarker for NPC. Based on our findings, the cutoff value of EBV DNA for prognosis of NPC was 262.7 copies/ml. The novel combination biomarker of EBV-DNA and PNI was a promising biomarker to predict NPC survival and may someday guide treatment options.

## Methods

### Participants

During the period from October 2015 to December 2018, 493 NPC patients who met the inclusion criteria below received standard anticancer treatment at Sichuan Cancer Hospital. The following are the inclusion criteria of this retrospective study: (1) pathologist initially diagnosed NPC. (2) Patients aged 18–70 years old at diagnosis. (3) Received standard chemoradiotherapy according to the national guideline of NPC. (4) Prior to anticancer treatment, patients had thorough examination and laboratory tests, including EBV DNA, blood routine and biochemistry. (5) With regular follow-up after treatment. Patients who were diagnosed with two or more types of malignant cancer at the same time, as well as those who received non-standard treatment or had missing follow-up information, were excluded from this study. Patients’ clinical stages were restaged according to the 8th version tumor–node–metastasis (TNM) staging system of the Union of International Cancer Control and the American Joint Committee on Cancer (UICC/AJCC).

This respective study was approved by the institutional Ethics Committee for Medical Research and New Medical Technology of Sichuan Cancer Hospital (SCCHEC‑02‑2019‑10), and the research process followed the 1964 Helsinki promulgated.

### Data collection

We obtained basic clinical pathologic information, age, gender, clinical stage, treatment summary, and laboratory results from medical record system. The nutritional indicator (including serum albumin and lymphocyte count) and plasma EBV DNA were necessary. Examination should be completed within one week prior to anticancer treatment. The nutritional indicator PNI and neutrophil-to-lymphocyte ratio (NLR) were calculated using the formula below.

PNI (prognostic nutritional index) = serum albumin (g/L) + 5 × lymphocyte count (10*9/L).

NLR (neutrophil-to-lymphocyte ratio) = neutrophil count (10*9/L)/lymphocyte count (10*9/L);

### Quantification of plasma EBV DNA

2–3 ml of peripheral venous blood samples were collected and centrifuged at 3000 r/min for 10 min. Plasma was collected for EBV DNA quantification by quantitative real-time PCR(q-PCR) method, which was mainly targeted the BamHI-W region of EBV genome. The unit of plasma EBV results were copies. The procedure was performed according the manufacture of EBV viral nucleic acid amplification kit (Sheng Xiang, Hunan, China). Briefly, 100 ul plasma sample and 100 ul commercial concentration agent were blended and centrifuged at 12,000 r/min for 5 min. Then supernatant liquid was discarded, and 100uL release agent was loaded. 10 min later, the q-PCR reactions were consisted with 10ul extracted DNA and 40 ul PCR- mixture. PCR amplification parameters were set as 50 °C for 2 min, initial denaturation at 95 °C for 5 min, 45 cycles of denaturation at 94 °C for 15 s and extension at 57 °C for 3 s, and 37 °C for 30 s. Fluorogenic amplifications were performed with ABI7500 Real-Time PCR System (Thermo Fisher Scientific, ABI, America). The results were showed as the copies of EBV DNA per milliliter of plasma.

### Treatment

According to the national guideline of NPC^46,47^, patients in this study were received normative treatment options based on their clinical stage. Patient in stage I-II received radiotherapy alone or concurrent chemoradiotherapy (CCRT) (for those with N +), while those in stage III-IVA received CCRT or neoadjuvant chemotherapy plus CCRT chemoradiotherapy (especially for those with high risk factors such as T4, N3, and high EBV DNA load). Patients suffered with IVb (with distant metastasis) received systemic cisplatin-based chemotherapy based comprehensive treatment. 4–6 cycles of GP (Gemcitabine: 1000 mg/m^2^d1, d8; Cisplatin: 80 mg/m^2^d1) or TPF (Docetaxel: 60–75 mg/m^2^, d1; Cisplatin: 60–75 mg/m^2^, d1; fluorouracil: 600–750 mg/m^2^d1-5) chemotherapy before irradiation to the nasopharynx or metastases. For patients underwent CCRT, chemotherapy of at least 7 times of weekly regimen (cisplatin: 40 mg/m^2^) or at least 3 times of 3-week regimen (cisplatin: 80–100 mg/m^2^) be presented while receiving radiotherapy. For NPC at stages III-IVA with CCRT only (except for T3N0M0), adjuvant chemotherapy for 2–3 cycles (interval 3–4 weeks) in total were conducted after CCRT. 70 Gray or greater dose by Intensity-modulated radiotherapy (IMRT) were prescribed to the primary tumor and 60–70 Gray dose to the metastatic cervical lymph node, the subclinical volume was prescribed 50-54 Gy or greater. The radiotherapy details and relative parameters were consistent with previous work we published^48^.

### Follow-up

Patients were scheduled to receive regular follow-ups for every three months for the first two year following treatment, every six months for the third to fifth year, and annually after that. The OS and PFS were calculated from the start of treatment to the date of event or last follow-up which were in line with our previous work^10^.

### Statistical analysis

Data from the normal distribution were expressed as average ± standard deviations, while data from non-normal distribution were shown as median (first quartile, third quartile). Statistical analyses were operated on SPSS 26.0 statistical software. Sperman rank correlation test was used to explore the correlation between EBV DNA and clinical indicators. The Kruskal–Wallis test was used to determinet the difference of EBV DNA in each subgroup. Survival rate was calculated by Kaplan–Meier method. The log-rank test was used to examine the difference in survival between each subgroup. Multivariable analysis to explore the prognostic factors were performed by COX regression model. Cutoff finder (https://molpathoheidelberg.shinyapps.io/Cutoff%20Finder_v1/) was applied to select the best EBV DNA and PNI cutoff value for OS. ROC and survival curve were plotted on SPSS software. MedCal statistical software was used to compare the AUC of different ROCs. A *P* value less than 0.05 was considered as statistically significant.

### Ethics approval and consent to participate

The study was approved by the ethics committee of the the Institutional Ethics Committee of Sichuan Cancer Hospital (SCCHEC-02-2019-10) and carried out in accordance with relevant guidelines and regulations. All patients included in this study signed informed consent to the treatment protocol statements.

## Supplementary Information

Supplementary Legends.

Supplementary Figure S1.

Supplementary Table S1.

Supplementary Table S2.

## Data Availability

Data are available from the corresponding author upon reasonable request.
