# Supplementary material for: A promising predictive biomarker combined EBV NDA with PNI for nasopharyngeal carcinoma in nonendemic area of China
Source: Sci Rep. 2023 Jul 20;13:11700. doi: 10.1038/s41598-023-38396-z (PMC10359455; doi:10.1038/s41598-023-38396-z)
Supplement: Supplementary file 1 — Supplementary Legends. [file 41598_2023_38396_MOESM1_ESM.pdf]

Supplementary Figure Legend

Figure S1. The prognosis of EBV-DNA≤262.7Copies/ml group and EBV-DNA>262.7copies/ml in locally advanced stage (LA, N=428), M0 (N=456), and M1 (N=37) patients. Figure S1A, C, E: The 5-year OS of patients with EBV-DNA ≤262.7copies/ml and EBV-DNA>262.7copies/ml group. Figure S1B, D, F: The 5-year PFS of patients with EBV-DNA ≤262.7copies/ml and EBV-DNA>262.7copies/ml group.
