# Supplementary figures and images for: A promising predictive biomarker combined EBV NDA with PNI for nasopharyngeal carcinoma in nonendemic area of China
Source: Sci Rep. 2023 Jul 20;13:11700. doi: 10.1038/s41598-023-38396-z (PMC10359455; doi:10.1038/s41598-023-38396-z)

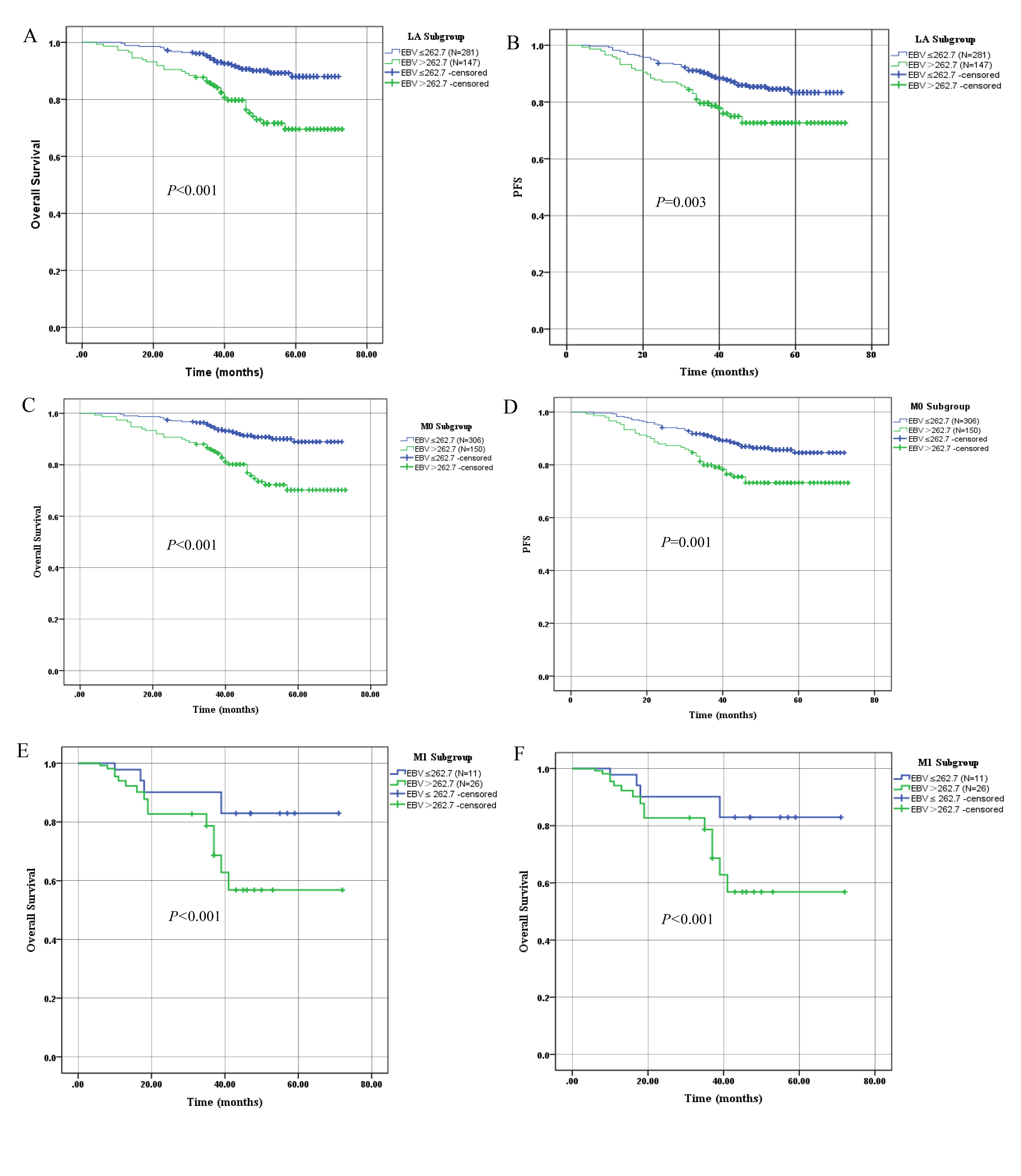

Supplement: Supplementary file 2 — Supplementary Figure S1. [file 41598_2023_38396_MOESM2_ESM.tif]
