# Supplementary material for: A promising predictive biomarker combined EBV NDA with PNI for nasopharyngeal carcinoma in nonendemic area of China
Source: Sci Rep. 2023 Jul 20;13:11700. doi: 10.1038/s41598-023-38396-z (PMC10359455; doi:10.1038/s41598-023-38396-z)
Supplement: Supplementary file 3 — Supplementary Table S1. [file 41598_2023_38396_MOESM3_ESM.pdf]

Table S1. The AUC of different prediction models.

Variables	5-year OS			5-year PFS		
	**AUC**	**SE [a]**	**95% CI**	**AUC**	**SE [a]**	**95% CI**
PNI	0.628	0.024	0.583 - 0.670	0.586	0.025	0.542 - 0.630
TNM	0.675	0.029	0.632 - 0.716	0.655	0.028	0.611 - 0.697
EBV	0.659	0.029	0.615 - 0.701	0.621	0.028	0.577 - 0.664
PNI+TNM	0.574	0.031	0.529 - 0.618	0.584	0.029	0.539 - 0.628
EBV+PNI	0.709	0.028	0.667 - 0.749	0.651	0.029	0.608 - 0.694
EBV+TNM	0.724	0.029	0.682 - 0.763	0.684	0.029	0.641 - 0.725

[a] DeLong et al., 1988
