# Supplementary material for: A promising predictive biomarker combined EBV NDA with PNI for nasopharyngeal carcinoma in nonendemic area of China
Source: Sci Rep. 2023 Jul 20;13:11700. doi: 10.1038/s41598-023-38396-z (PMC10359455; doi:10.1038/s41598-023-38396-z)
Supplement: Supplementary file 4 — Supplementary Table S2. [file 41598_2023_38396_MOESM4_ESM.pdf]

Table S2. The comparison of different prediction models' AUC ROCs

**OS**	**EBV** vs **PNI**	**EBV** vs **TNM**	**PNI** vs **TNM**	**EBV** vs **EBV+PNI**	**EBV** vs **EBV+TNM**	**EBV+TNM** vs **EBV+PNI**
△AUS	0.031	0.016	0.048	0.050	0.065	0.014
Z	0.875	0.453	1.256	2.740	2.934	0.525
P	0.381	0.651	0.209	**0.006**	**0.003**	0.600
**PFS**	**EBV** vs **PNI**	**EBV** vs **TNM**	**PNI** vs **TNM**	**EBV** vs **EBV+PNI**	**EBV** vs **EBV+TNM**	**EBV+TNM** vs **EBV+PNI**
△AUS	0.035	0.034	0.069	0.030	0.063	0.033
Z	1.021	1.043	1.956	1.642	3.117	1.319
P	0.307	0.297	**0.050**	0.101	**0.002**	0.187
